# Predicting complex phenotype–genotype interactions to enable yeast engineering: *Saccharomyces cerevisiae* as a model organism and a cell factory

**DOI:** 10.1002/biot.201300138

**Published:** 2013-08-23

**Authors:** Duygu Dikicioglu, Pınar Pir, Stephen G Oliver

**Affiliations:** 1 Cambridge Systems Biology Centre and Department of Biochemistry, University of Cambridge, CB2 1GA, Cambridge, UK; 2 Babraham Institute, Babraham Research Campus, CB22 3AT, Cambridge, UK

**Keywords:** Engineered strains, Fitness, Growth control, Human diseases, Yeast

## Abstract

There is an increasing use of systems biology approaches in both “red” and “white” biotechnology in order to enable medical, medicinal, and industrial applications. The intricate links between genotype and phenotype may be explained through the use of the tools developed in systems biology, synthetic biology, and evolutionary engineering. Biomedical and biotechnological research are among the fields that could benefit most from the elucidation of this complex relationship. Researchers have studied fitness extensively to explain the phenotypic impacts of genetic variations. This elaborate network of dependencies and relationships so revealed are further complicated by the influence of environmental effects that present major challenges to our achieving an understanding of the cellular mechanisms leading to healthy or diseased phenotypes or optimized production yields. An improved comprehension of complex genotype–phenotype interactions and their accurate prediction should enable us to more effectively engineer yeast as a cell factory and to use it as a living model of human or pathogen cells in intelligent screens for new drugs. This review presents different methods and approaches undertaken toward improving our understanding and prediction of the growth phenotype of the yeast *Saccharomyces cerevisiae* as both a model and a production organism.

## 1 Introduction

Yeast has successfully been used and manipulated to be used as a model organism in fundamental and applied research. The advances in genome-wide screening approaches facilitated the use of this model for medical and medicinal research. Being a genetically well-defined and well-tractable organism that is amenable to gene mutations, disruptions, or gene marking as well as gene-dosage effects renders yeast attractive in the study of disease processes such as the regulation of eukaryotic cell division or the elucidation of events leading to apoptosis [1].

Since systems biology aims to achieve a quantitative phenotypic description of a biological system, advances in this particular field through the high-throughput analysis of cell parts and genome-scale metabolic modeling have been incorporated in medical and medicinal biotechnology applications. The use of systems biology as an approach in “red” or “pharmaceutical” biotechnology initiated the efforts to achieve a systems level understanding of the microorganisms that are suitable for biomedical research. Systems biology, synthetic biology, and evolutionary engineering research all influence biomedical research in an interactive manner to accelerate the path toward understanding the connection between the genotype and the phenotype. Fitness has been studied extensively to understand this complex relationship. Moreover, the influence of environmental effects further complicates the objective of understanding cellular mechanisms leading to healthy or diseased phenotypes [2].

**Figure 1:**
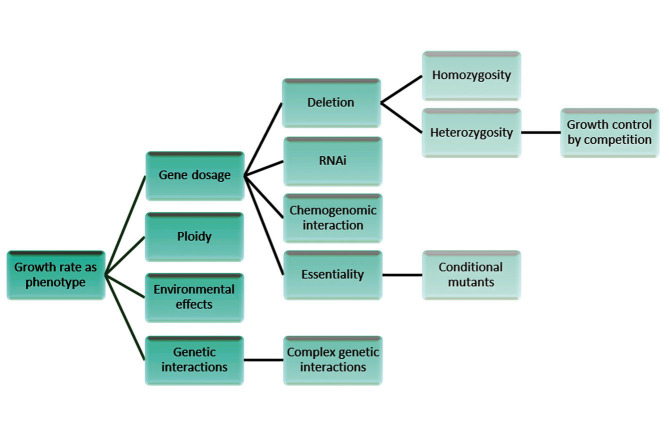
An overview of the efforts taken toward achieving a full understanding of the genetic and physiological control of cell growth. Studies were conducted to investigate the effect of gene dosage, ploidy, environmental factors, and genetic interactions on improving our understanding of growth rate as a phenotype.

Prediction of an optimal growth rate as a measure of growth phenotype could be achieved through various mathematical metabolic optimization methodologies making use of constraint-based formalisms [3]. This review focuses on different methods and approaches that are followed for understanding and predicting the growth phenotype in yeast as a model organism (Figure 1). The impact of strain-to-strain differences, such as auxotrophies and gene copy-number variation, on the growth phenotype will also be discussed in detail.

## 2 Cellular processes controlled by growth

The growth rate of a cell is described by the physiological activities taking place within the cell, such as the rate of biosynthesis of biomass precursors. However, the relationship between growth rate and physiological activities is not unidirectional, a range of cellular activities are in turn regulated by the growth rate itself. Before describing how growth rate is controlled, this section will provide an overview of the cellular processes that are under the control of growth. Early experimental evidence on the existence of this bi-directional dependency indicated that cell-size regulation is dependent on growth rate: McMurrough and Rose [4] cultivated yeast in chemostats under carbon and nitrogen limitation to identify the cell wall components. In addition to finding condition-dependent differences in cell-wall composition, they reported that cell size depended on the growth rate rather than on the limiting nutrient. Indeed, the mechanisms of cell size regulation by growth rate were revealed via microscopy-based single-cell measurements [5] and mathematical modeling [6]. Parallel studies were also carried out for the fission yeast *Schizosaccharomyces pombe* [7].

Most of the growth-regulated processes were identified after high-throughput techniques for the investigation of “omics” became available. A small number of benchmark studies have produced comprehensive lists of growth-controlled processes under nutrient limitation [8–11]. Castrillo et al. [8] demonstrated that the transcriptome, proteome, endo-, and the metabolome of yeast are regulated by growth rate in cells growing under four different nutrient-limiting conditions. For instance, at the transcriptomic and the proteomic levels, Gene Ontology (GO) analysis of regulated components indicated that protein synthesis pathways were up-regulated with increasing growth rate [8]. Individual analysis of measurements from each nutrient limitation demonstrated that regulation was often limiting-nutrient specific [11]. These studies also demonstrated that the transcriptome and the proteome of the cells were not strongly correlated; hence growth control could be at both the transcriptional and the translational level. In a similar study with yeast growing in fermenters under six different limiting nutrients, it was shown that the fraction of cells in G0/G1 phase of the cell cycle was linearly correlated with the growth rate, as were the levels of hundreds of transcripts [9]. Growth control of genes at different levels of oxygen availability and nutrient limitation reveal that metabolism-related genes were also growth-regulated, and this regulation was likely to be coordinated by transcription factors (TFs) whose target genes were enriched with those associated with ribosome biosynthesis, cell cycle, and stress response GO terms [10]. Metabolome profiles of yeast growing in fermenters indicated that the intracellular levels of one or more metabolites were correlated with the growth rate, and hence could be limiting, or determining, that rate. The set of limiting metabolites depended on the limiting nutrient in the growth medium [12]. All this evidence indicated the existence of a multi-level and inter-connected regulation of cellular processes by the growth rate.

As we mentioned above, growth rate not only regulates other cellular processes but is also regulated by cellular processes and environment. Slavov and Botstein observed growth rate signals to be a function of the abundance of essential nutrients, which (in turn) regulate fermentation/respiration, the growth rate response, and the cell division cycle [13, 14]. The coupling between oxygen availability, transcriptional changes leading to growth rate differences and the environmental stress response was discussed previously [15]. Airoldi et al. [16] computationally predicted the strong up-regulation of growth as a response to the absence of appropriate nutrients. Boer et al. [17] suggested that starving yeast auxotrophs wasted glucose as a consequence of the failure of conserved growth control pathways. Furthermore, particularly strong concentration responses were observed for metabolites closely linked to the limiting nutrient in the metabolic network, e.g. glutamine in nitrogen limitation, ATP in phosphorus limitation, and pyruvate in carbon limitation. This indicated that a simple, but realistic, model involving the availability of these metabolites was adequate to account for cellular growth rate [12]. In their study of steady-state continuous cultures limited by one of six different nutrients (glucose, ammonium, sulfate, phosphate, uracil (Ura), or leucine (Leu)), Brauer et al. [9] reported a negative linear correlation between growth and a set of genes involved in peroxisomal functions, as well as a positive linear correlation between growth and a set of genes involved in ribosomal functions. Growth rate was also correlated with many genes associated with stress response and genes that are periodically expressed under conditions of metabolic cycling [11]. In addition, several studies focused on investigating which growth-associated processes were involved during the different phases of batch growth. Such growth-phase-regulated processes include quiescence [18–20], respiratory/fermentative growth/diauxic shift [21, 22], starvation [23, 24], phase of the cell cycle [25, 26], and cell size [27]. These were also identified in high-throughput analyses of cellular components, mostly from transcriptome data.

### 2.1 Essential genes: Can we compensate for extreme metabolic growth control?

In this section, we will review a few of the pioneering studies regarding essential genes and how our understanding of growth control by such genes has evolved. Essential genes encode proteins that are indispensable for cellular growth; hence such genes can be viewed as having “extreme control” on growth. A subset of essential genes encodes the metabolic enzymes, which catalyze biosynthesis of the essential metabolic precursors of biomass. In the absence of these genes, the organism has to be supplemented for the corresponding essential nutrient; otherwise growth cannot be achieved.

Strains with disrupted essential metabolic genes are often called auxotrophic, as opposed to strains that are self-sufficient (prototrophic). Following the pioneering work on yeast life cycle and genetics by Lindegren [28] and the appearance of quantitative data on nutritional control of growth in the literature [29], mutations causing auxotrophy were exploited in order to develop methods for strain selection [30] to isolate transformants constructed using genetic engineering strategies.

Efforts to track down the pedigree of yeast laboratory strains were initiated in 1980s as heterogeneities between strains used in different laboratories were identified [31] and the need for standard strains emerged (see [32] for a review on strains). Prior to the yeast genome-sequencing project, auxotrophic strains isogenic to S288c were constructed and distributed to many laboratories [33, 34], and they were used as the background strain for yeast deletion mutant libraries [35, 36]. The *HO* locus [37, 38], which encodes a gene that is not transcribed in diploid cells, but is essential for mating-type switching in haploids, was selected as a neutral locus for inserting antibiotic marker cassettes in diploid strains in order to control for marker effects and generate a standard reference strain [39].

However, the use of auxotrophic markers in standard laboratory strains came under scrutiny following the establishment of more sensitive tools to detect fine differences in growth rates and metabolic activities. Even when supplementary nutrients are present in excess, auxotrophic strains were reported to have altered metabolism and growth patterns, providing evidence that the impact of auxotrophic markers could not be simply compensated by supplementing the growth medium [40–44]. The yeast research community responded to this problem by constructing novel prototrophic yeast strains, with the aim of overcoming the bias introduced by the auxotrophic background of S288c. One such contribution was the construction, and inter-laboratory characterization, of the Yeast Systems Biology Network (YSBN) strains [45]. YSBN strains were derived from S288c via the integration of the *URA3* gene into its genome and the insertion of antibiotic resistance markers in the *HO* locus and they were compared with the prototroph CEN-PK113-7D, which was previously investigated in an inter-laboratory experiment [46] in terms of its growth characteristics and high throughput transcriptional and metabolomic profiles under different growth conditions. YSBN2 and CEN-PK113-7D were shown to have different physiological parameters, and the differences in their transcriptome and metabolome profiles were related to protein and lipid synthesis pathways, indicating that the variation in the metabolic network of the two prototrophic strains may contribute the most to the differences that were identified between them.

Recently, a systematic study on the effects of Leu^–^, histidine (His^–^), Ura^–^, and methionine (Met^–^) auxotrophies on yeast growth and aging was conducted and a prototrophic library of deletion mutants was built by the re-introduction of genes on a mini-chromosome to complement the auxotrophic markers [43]. The use of *HO* locus in YSBN strains and the use of a mini-chromosome in the prototroph library may still introduce metabolic and growth-related biases in these new strains although they are a step towards the complete elimination of systematic metabolic biases in high-throughput systems biology studies. The lesson learned from the study of auxotrophic strains was that the essential genes would have control over the highly inter-connected metabolic network in, as yet, unpredictable ways.

Gene essentiality is context dependent; hence a gene may be essential only under a given set of growth conditions, but not under other conditions. Easy detection of the presence or absence of colony growth under the investigated conditions was exploited in early studies that were conducted to identify genes that would be responsible for relevant functions. Indeed, many families of genes were identified following the discovery of their condition-dependent essentiality in yeast, including the *SIR2* family with their essentiality in mating [47], the nuclear petite (*PET*) genes with their essentiality for respiration [48], and the target of rapamycin (*TOR*) genes for being targets of rapamycin [49].

Context dependency of essentiality can be extended from growth media to other growth parameters such as temperature. Tools for investigating essential gene-related phenotypes was initiated with the isolation of temperature-sensitive mutants of yeast [50]. Such mutants have no growth deficiency at a normal temperature range, but are sensitive to temperatures outside that range. Recently, a collection of temperature-sensitive mutants covering 45% of all essential genes was built [51] and this collection was used for the chemical-genomic suppression and the quantitative analysis of phenotypes. Another collection, which covered ∼82% of the essential yeast genes, was the decreased abundance by mRNA perturbation (DAMP) collection [52], which was demonstrated to be a useful tool for the analysis of phenotypes related to essential genes. The PhenoM database was constructed to store phenotypic data generated by such efforts [53].

## 3 Computational prediction of gene essentiality

Computational prediction of gene essentiality is a relatively new concept. Such computations were carried out using stoichiometric models of metabolism. Stoichiometric metabolic models of yeast were initially constructed to simulate only the central metabolic pathways and predict the carbon fluxes directed to products such as ethanol or glycerol [54]. Availability of genome sequences allowed the identification of genes likely to encode enzymes. This was followed by the reconstruction of genome-wide metabolic models, which had hundreds of metabolites and reactions representing uptake and utilization of nutrients, and the production of biomass and of various other products that were secreted into the growth medium. The first genome-wide model of *Saccharomyces cerevisiae* became available in 2003 [55], and the first task for which it proved useful was the prediction of essential genes via simulations of cell growth that involved treating the network as a constraint-based linear optimization problem [56]. However, growth simulations made using the genome-wide metabolic models are not 100% accurate; the predictions have a certain burden of false positives and negatives. The negatives stem from the fact that the models are not yet complete, and hence not every route to the biosynthesis of biomass precursors is represented by the models. This kind of imperfection in the model causes false negatives and the gene is predicted to be essential although, in fact, it is non-essential. Furthermore, the directionality of the reactions often lack experimental evidence and, in rare instances, some reactions may even be incorporated into the model with no experimental evidence, causing false positives and this causes a gene to be predicted as non-essential even though it is essential. Genome-wide stoichiometric models are updated frequently by the community, with the aim of increasing their accuracy of prediction (http://sourceforge.net/projects/yeast/files/).

Prediction of the essentiality of genes under different growth conditions can be analyzed by modifying the nutrient uptake constraints that apply to the model and by incorporating the known regulatory events such as carbon catabolite repression or response to stress into the model via constraining the flux through the relevant reactions. Growth in minimal, complete, or rich media with a variety of chemical compositions can also be simulated using the chemical composition of the growth medium as a set of constraints on the model. Simulation results are then compared with the experimental results and the model refined and improved accordingly. An example of such a study is that carried out by Snitkin et al. [57], in which the essentiality of 465 genes was tested in 16 types of growth media, and the improved model was used to test alternative routes of raffinose utilization by yeast. Validation of metabolic models by predicting gene essentiality is a routine procedure and the frequency of true positives is a measure of the network’s quality. Other criteria, such as success in predicting the growth patterns under aerobic/anaerobic conditions, or the prediction of epistatic interactions between genes, are also used for the validation of models.

While metabolic models are improving their accuracy in predicting gene essentiality, only genes involved in metabolism are represented in these models. Prediction of essentiality of non-metabolic genes under a given set of growth conditions requires the integration of data from heterogeneous sources (often protein–protein interaction networks) and more sophisticated computational methods such as machine learning [58–61].

## 4 High-throughput phenotypic analysis of strains growing in competition

Most of the (6000 genes in the yeast genome are not essential under a given growth condition; however, growth may be impaired or improved in the absence of some of the genes. Growth impairment or growth deficiency may be an indication that the protein products of these genes are involved in the processes that control growth. Soon after the yeast genome became available, deletion mutant libraries of the non-essential genes were constructed. The utilization of these libraries was shown to be a convenient and standard tool for many small-scale phenotypic studies covering a limited number of mutants, as well as genome-wide studies [62]. Currently, more than 500 publications reporting large-scale phenotypic analyses are listed in the Saccharomyces Genome Database (SGD) Literature pages (http://www.yeastgenome.org/cache/genome-wide-analysis.html). Any possible errors or biases in the libraries are being iteratively corrected by the yeast community [63, 64].

Phenotype analyses integrated with metabolomics or chemical treatment revealed unexpected information about yeast physiology. For instance, even when there was no growth phenotype, gene deletions were shown to have a detectable effect on the metabolism of the mutants [65]. Hillenmeyer et al. concluded that 97% of yeast genes were required for optimal growth under at least one growth condition, after screening the deletion mutants under 1184 chemical genomic conditions [66]. Strains in the yeast deletion libraries bear antibiotic marker cassettes with unique molecular barcodes, making it possible to identify and quantify each deletion strain during growth in a competition pool [67, 68]. Tag arrays were designed for the hybridization of the barcodes in deoxyribonucleic acid (DNA) extracted from the pooled population [69] and, more recently, next-generation DNA-sequencing methods [70] have been used to identify the different mutant strains in a pool and to quantify their growth phenotypes.

Although phenotype screenings of individually grown mutants either as colonies or liquid cultures has provided an invaluable source of information about condition-dependent gene function, in this section we will limit our discussion to phenotype screening by monitoring growth in competition pools. Phenotype screening was often carried out to identify mutants with phenotypes during the limitation or absence of a nutrient. Grape juice, a natural growth medium that yeast has evolved to utilize, was reported to be poor in nitrogen sources [71]. Deletion mutants of genes with functions related to protein degradation pathways were among the mutants that had fitness advantage (haploproficient) in grape juice fermentations [72] and similar results were previously reported in the fermentation of the heterozygous deletion pool in competition both in nitrogen-limited synthetic medium and in grape juice [73, 74]. This indicated that, under nitrogen limitation, keeping the existing proteins rather than degrading them could be a selective advantage. Inositol is an essential component of various secondary messengers and structural lipids in the cell. Competition of mutant libraries in defined medium without inositol identified hundreds of mutants with growth phenotypes and the results indicated a link between inositol metabolism and the stress response [75]. Phenotypes of mutants grown in competition under Leu or phosphate starvation shed light on the genes that were required for the survival of starved cells [76, 77]. Zinc, an essential trace element that plays a role in both the structure and interactions of many proteins, was studied in similar competition experiments of all non-essential gene deletion mutants and hundreds of genes were found to be important for fitness under zinc-limited growth [78].

The “marginal fitness hypothesis” states that genes with no apparent deletion phenotype may also have growth control and that the control which they exert can be measured with high-sensitivity techniques, one of which was growth of multiple strains in competition in the same growth medium [79]. The marginal fitness hypothesis is in accordance with principles of metabolic control analysis (MCA) [80], which provides a mathematical formulation of flux control by all the components of a system. Although the main focus of MCA is the control that a flux through a reaction exerts on the metabolic pathway, the concept applies to any flux leading to a product, which can be biomass formation, growth rate, or relative growth rate [74, 81]. Hence, the phenotype profiles of gene-deletion mutants can ultimately be used in constructing mathematical models of growth control by genes, although the large number of genes playing a role in growth control and the necessity for measurements at multiple levels of gene dosage makes this task impractical for the time being. The accuracy of high-throughput phenotype measurements has also been discussed from both an experimental and a bioinformatics point of view [82]. Additionally, genetic interactions between genes are rare but they have a detectible impact on phenotype, with a wide range of dosage effects from causing only a few per cent change in growth phenotype to the extreme case of synthetic lethality (see the section below on Gene Interactions) (specify section number). Other cellular components like non-coding ribonucleic acids (RNAs) [83] or metabolites [51] also contribute to the control of flux. Therefore, a model built on growth control of single genes may be unrealistic in predicting phenotypes except for isolated cases. Nevertheless, the phenotypes of gene deletion mutants relative to those of a mutant pool would provide insight regarding cellular processes having a large growth control under the investigated conditions. Often such datasets have been integrated with other sources of data in order to build network-based models [61, 84–86].

## 5 Growth rate and dosage balance theory from an evolutionary perspective

Understanding growth is crucial since growth rate as a variable or as a parameter has a central role in evolutionary biology, functional genomics, and systems biology [87]. Growth phenotypes, the effects of mutations, and theories of dominance have been the topic of discussion on a conceptual and theoretical basis since the early 20th century. In very early discussions, Fisher suggested that many mutations had already occurred and the observed wild-type phenotypes were in fact those mutations that survived and that complete dominance would generally be regarded as a product of selective modification. If a heterozygote was at any appreciable disadvantage with respect to the wild type, it would be observed at a much higher frequency than its homozygote which would be anticipated to have an even lower viability, thus the heterozygote would constitute a higher fraction of the population [88, 89]. From an evolutionary perspective, Wright discussed the physiological aspects of dominance theory, stating that survival was closely linked with the mutational change being beneficial for the organism or not [90]. Kacser and Burns explained the dominance of enzymes in a metabolic pathway using Wright’s definitions of genetic dominance and a gene’s phenotypic impact was effected through its expression and the extent of control its enzyme exerts at a particular step in a metabolic pathway, also establishing the concept of MCA [91]. A more recent discussion on genetic dominance categorized alleles as dominant, recessive, or semi-dominant and took haploinsufficiency into consideration. Recessivity of the majority of the mutations to wild type identified recessiveness as the default state and dominance as representing a minority or exceptional state. The molecular mechanisms causing dominance were classified into different categories including reduced or increased gene dosage and expression as well as protein activity, dominant-negative effects, altered structural proteins, toxic protein alterations, and gain-of-function mutations [92].

Haploinsufficiency is most frequently associated with compromised survival of the individual either as a multicellular organism or as a member of a single-celled population. Haploinsufficiency, a case of genetic dominance apparent in the presence of only a single allele of a normally diploid locus was previously discussed specifically for the TF genes. The synergistic interactions among TFs were used to explain a proportion of the haploinsufficiency cases and a deterministic mass-action model was constructed to explore haploinsufficiency in systems involving simple TF complexes. Halving the input of a complex subunit was further shown to induce either proportional or more-than-proportional changes in the total output of the macromolecular complex and haploimbalance was introduced to account for this concept [93]. Papp et al. [94] tested this notion within the context of the balance hypothesis and showed, for the first time, that the under- or over-expression of a member of a protein complex or single-gene duplications caused reduced fitness in yeast. Imbalance was shown to have deleterious effects and it was also demonstrated that interacting proteins were more frequently co-expressed than random pairs, also indicating that they either both go through duplication or both remain as solo copies. The findings were congruent with former theories, which indicated that genetic dominance was a by-product of physiology and metabolism and did not simply represent a masking of the deleterious effects of mutations. Further study showed that genes encoding members of protein complexes need not only to maintain the abundance ratios of their products (to prevent severe phenotypic penalties, including death), but also have a tendency to be co-localized in the genome, i.e. within 10–30 kb (kilobases) of each other [95]. However, the cellular levels of the sub-units of protein complexes were shown to be not necessarily maintained at their optimal values. For instance, the genes encoding members of the proteasomal complex were identified as being haploproficient under nitrogen limitation, although theory would predict growth deficiency due to dosage imbalance [74]. Another study focusing on dosage imbalance in *S. cerevisiae* investigated the cell cycle genes and the robustness or fragility of the metabolism in response to over-expression of single genes. The combinatorial “genetic tug-of-war” experiments that were conducted indicated that fragility arising from dosage imbalance could be masked by other genes but that, in situations where masking could not be achieved, dosage imbalance would cause fragility [96].

Current studies on understanding human disease states led researchers to think that there was increasing evidence that heterozygous null alleles led to more and more human diseases, displaying the importance of haploinsufficiency in disease. Numerous haploinsufficient genes that were identified in yeast indicated that a 50% reduction in gene activity not yielding wild-type phenotypic characteristics showed that the former assertions that most mutations were recessive did not hold [97]. The possibility of co-duplication of unlinked dosage-sensitive genes or small subsets of genes was ruled out in the light of these findings and the presence of the paralogs of dosage-sensitive genes indicated the occurrence of a whole-genome duplication (WGD), and led to an investigation of gene-dosage balance and haploinsufficiency. The impact of polyploidy on the evolution of networks was considered to be much more important than the cumulative effect of individual gene duplications. The fact that the diploid cell volume was approximately twice that of haploid cell volume was also shown to support the notion that large-scale duplications took place in order to produce paralogous pathways. Moreover, the paralogs were suggested to diverge in sequence and pattern of expression in order to prevent any stoichiometric interference and thus avoid imbalances post-duplication [98]. Birchler et al. [99] further investigated the potential contribution of regulatory balance to the control of phenotypic characteristics. They discussed evidence for the role of regulatory imbalance in disease phenotypes, which can involve the aneuploidy of genes specifying regulatory components, such as transcriptional regulators, signal transducers; haploinsufficient genes encoding structural proteins or sub-units of protein complexes, and the human counterparts of such genes. They concluded that changes in the levels of individual members of a regulatory complex would affect the workings of the whole complex and the outcome of this effect would manifest itself in the corresponding phenotype [99].

### 5.1 Competitive growth in the fermentation of yeast heterozygote populations

In order to be able to assign biological meaning to genomic data, researchers aim to understand the contributions of many sequence variants to phenotypic variation both within and between species. Phenotypic analysis of mutants was reported as a useful approach in determining gene function and identifying the genetic variants that would be responsible for complex traits. For this purpose, gene function was altered systematically through deletions, insertional mutagenesis, or RNA interference and attempts were made to quantify the reduction or increase in fitness of the cell in response to these alterations [100]. A recent discussion focused on the studies that were conducted over the past decade in which high-throughput yeast deletion mutants were systematically analyzed, which was expected to yield a major increase in our understanding of dominance from an evolutionary perspective. A significant issue, which was addressed, was that the results of such studies needed to be treated cautiously, especially when the identified growth defect was small to moderate since errors in analysis and/or measurement and could lead to mistakes in the prediction of dominance [82].

One of the earliest attempts taken toward the quantification of genetic impacts on fitness was the study of the yeast orthologs of mitochondrial human disease/disorder genes. In this first high-throughput study of screening genome-wide heterozygous yeast mutants, the effect of using fermentable or non-fermentable carbon sources in the fermentation medium was investigated by growing the mutants in competition pools. The study reported very high selection of mitochondrial genes in the yeast deletion screen, whose orthologs would serve as candidates for mutational screens in Mendelian and complex mitochondrial disorders in humans [19]. An analysis that resulted in the complete dissection of a quantitative trait locus (QTL) in *Saccharomyces cerevisiae* used a combination of two techniques: genome-wide mapping and reciprocal hemizygosity analysis; it provided important information regarding heterosis and the increased fitness of heterozygotes compared with the corresponding homozygotes [101]. Fitness profiling was also used to identify cases of haploinsufficiency in yeast resulting from heterozygosity caused by the loss-of-function of an allele. The competitive growth of the yeast mutants in heterozygote pools revealed that the main reason for haploinsufficiency in yeast was the lack of sufficient protein production. Heterozygosity can also result in impaired ratios of the members of protein complexes and so cause haploinsufficiency as predicted by the dosage balance hypothesis, with the high throughput competition results verified by individual growth assays [102].

The control of growth by high-flux-control genes was investigated in yeast through competition assays conducted in continuous cultures, which allowed fermentation under controlled conditions [103]. The control of flux was evaluated via the identification of genes displaying haploproficient and haploinsufficient growth profiles in competitive cultures of yeast. The study reported that haploinsufficient genes are associated with the determination of polar cell growth and were over-represented on chromosome III (the mating-type chromosome) and haploproficiency to be a phenotype that was more likely to be specific to a particular environmental context [74]. Another study focusing on the genetic control of growth rate in yeast employed a particular setup in which the hemizygous diploid yeast deletants were grown at or close to the maximum specific growth rate in nutrient-limiting or nutrient-sufficient conditions. Among the high-flux-control genes, the haploproficient subset was enriched for processes involving cell cycle and genome integrity, whereas the genes constituting the haploinsufficient subset were involved in gene expression. The results of this study indicated that the control of growth rate in yeast represented a trade-off between the selective advantages of rapid growth and the need to maintain the integrity of the genome [81].

The stress response of the heterozygous mutant pool of *S. cerevisiae* was investigated in another recent study by exposing the mutant pool to an unfavorable environmental condition, starvation, in order to determine the role of genetic heterogeneity in environmental fitness. The results indicated the importance of cellular recycling mechanisms in maintaining cell viability [77]. Another analysis on stress-induced fitness differences was conducted on the heterozygous yeast deletion mutant pool competitively grown under hypothermic stress and the homozygous yeast deletion mutant pool competitively grown under hyperthermic stress. The integration of the results from these analyses with GST pull-down experiments revealed physical interactions between general transcription, ribosome biogenesis, and guanosine triphosphate (GTP)-binding proteins and Hsp90p, which was used as an indicator to monitor hypothermic and hyperthermic stress responses [104].

Growth competition was also investigated in the fission yeast, *Schizosaccharomyces pombe*, although to a very limited extent. The availability of a heterozygous diploid deletion mutant library in *Sz. pombe* enabled the determination of fitness in the fission yeast, which is (so far) the only other eukaryote for which a comprehensive gene-deletion library exists. The haploinsufficient and haploproficient genes were determined and compared with those in *S. cerevisiae* and the results indicated that the control of eukaryotic cell control could be mediated by specific ribosomal proteins and RNA polymerase subunits [105].

Investigating the increase in gene copy number also proved a worthwhile route to phenotypic analysis in addition to the reduction in gene copy number. Phenotypes of yeast strains that overexpress genes can reveal unexpected fitness advantages or disadvantages. In classical metabolic control theory, enzymes were assumed to have a saturation point, beyond which they have no flux control [91]. However, the overexpression strains of yeast grown in competition indicated that genes with increased dosage would have flux control, and that the flux control could either be negative or positive [106, 107]. These recent studies in *S. cerevisiae* and *Sz. pombe* showed that haploproficiency was observed even in populations that were cultivated at their maximum growth rates in nutrient-sufficient environments.

Mutant libraries with tags integrated into the genome have proved to be a very important tool in yeast research and, recently, a TagModule system based on this tool was developed as a template for tagging the mutants of other organisms [108]. Furthermore, a bar-coded gene overexpression collection, BarFlex, was constructed recently with the aim of extending gene interaction screens to the overexpression of genes [109].

### 5.2 Phenotype analysis for drug discovery

Drug discovery, often referred to as “red biotechnology”, is one of the major fields of biotechnological applications. Determining the mode of action of drugs is an important step in drug discovery and yeast was used as a simple model organism in which drug targets could be discovered in a high-throughput manner [36, 110]. These so-called chemogenomic screens were facilitated by the use of phenotypic analysis of mutants growing in competition [111, 112] and this methodology has the advantage of providing a genome-wide screen without using vast amounts of the drug, which would usually be available in limited quantities. Screening the non-essential gene deletion collection with cytotoxic or cytostatic agents was used to suggest the mode of action for some of the agents tested, as well as the functions for unknown genes in the set [113]. The same methodology was used to identify the transporters of drugs, which were previously suggested to be transported via passive diffusion [114]. Targets of a microtubule-targeting agent [115] and genes that provided resistance to anti-microbial peptides from human saliva were also tested using the same methodology [116]. Yeast is one of the model organisms often used in aging research, and phenotype screens in yeast have also revealed genes with an effect on longevity [117].

Induction of haploinsufficiency via the introduction of drugs into the competitive growth environment of heterozygous diploid mutants of yeast cells was achieved in several studies in order to understand mechanisms of drug effectiveness and sensitivity on the lowering of copy number of genes. This method was employed for drug-target identification. A targeted set of 233 heterozygous deletion mutants of *S. cerevisiae* that has reduced copies of known drug targets was grown in the presence of the drug tunicamycin. The haploinsufficiency induced in the presence of the drug was identified and the sensitivity of these strains to the presence of tunicamycin was highly specific to treatment with tunicamycin only. This induced haploinsufficiency was suggested to be an aid to the elucidation of the mechanisms underlying heterozygous dominant autosomal disease phenotypes in higher eukaryotes [36]. A very comprehensive study on the effect of drugs on the decreased copy number of genes facilitated the screening of 78 therapeutically relevant chemicals across a genome-wide pool of tagged heterozygotes. This proof-of-principle study suggested the use of fitness profiling as a powerful approach for understanding the mechanism of action and the activity of the drugs on metabolism [118].

The effect of chromium toxicity was investigated by screening the heterozygous mutants of both the essential and the non-essential genes in yeast in the presence of chromium. The results of the study indicated the significance of proteasomal activity in chromium resistance. Oxygen-dependent messenger RNA (mRNA) mistranslation was observed to be induced during Cr exposure and Cr was shown to exhibit synergistic toxicity with paromomycin, which was reported to act via mistranslation [119]. Novel targets for nitrogen-containing bisphosphonates were identified in another study, in which genome-wide high-throughput screening of 5936 *S. cerevisiae* heterozygote barcoded mutants were competitively grown in the presence of risedronate, alendronate, and ibandronate. Furthermore, the results that were obtained from the yeast system were validated in a mammalian system taking a step further along the line toward drug discovery [120]. The effect of drug treatment on karyotype was investigated in *Candida albicans* with the aim of identifying the causal relationship between fluconazole resistance and acquired aneuploidy. *C. albicans* was shown to be more permissive of chromosome rearrangements and segregation defects in the presence of fluconazole [121].

Quantitative phenotyping of the complete heterozygote pool of the *S. cerevisiae* deletion collection was also investigated in a comparative study in which the performance of the microarray barcoding system was evaluated along with deep barcode sequencing. The comparative study was conducted in the presence and absence of drug treatment causing an inhibition in wild-type growth and the performance of deep barcode sequencing was claimed to outperform the profiling using barcode microarrays owing to the low percentage of sequencing errors and the improved sensitivity and dynamic range [70].

Another powerful technique that was used for competing strains in the same growth medium involved fluorescent labeling of the strains followed by growth in plate readers with fluorescent detection [122]. This method had the advantage of allowing the competitions between up to four yeast strains that bore enzymes from different organisms, such as the human dihydrofolate reductase (DHFR) and parasite DHFRs, to be carried out. The method was exploited to identify drugs which reduced the fitness of yeast strains that were dependent on the expression of an enzyme encoded by a gene from a human parasite for their growth, but not affecting the growth of a strain expressing the equivalent human enzyme. Similar work was done to identify genes relevant to calcium signaling [123].

### 5.3 Effect of multi-copy gene dosage in fitness phenotypes

The ancestor of *Saccharomyces cerevisiae* and its relatives went through a WGD approximately 100 million years ago. The yeast species that descended from this ancestor have twice the number of chromosomes as species that diverged from the Saccharomycetales prior to the WGD. However, the yeast that went through the WGD later lost nearly 90% of its duplicated genes. These observations led evolutionary biologists to the concept that there is a strong stabilizing selection in *S. cerevisiae* toward maintaining a diploid vegetative state. Tetraploid yeasts were reported to be less fit than their diploid relatives and they were reported to evolve to a diploid state in order to remove this selective disadvantage [124, 125]. Diploid cells were reported to remain stable due to the selection pressures of fermentation and increased ploidy would increase fermentative ability, especially in hybrid strains [126].

Haploids are, in general, smaller than diploids and their surface area to volume ratio is larger, hence their nutrient uptake is expected to be more efficient. Further, they have half the genome size of diploids, which should reduce the cost of cell division. Therefore, it may be hypothesized that haploids have higher fitness when compared to diploids under nutrient-limited condition (the nutrient limitation hypothesis; [127]). The nutrient limitation hypothesis was tested in order to explain the haploid/diploid cycle in yeast within the context of ploidy evolution. An isogenic series comprised of haploid, diploid, and tetraploid *S. cerevisiae* was constructed and their relative fitness was measured under different environmental conditions with a variation in the abundance of available nutrients as well as at optimal and non-optimal growth temperatures. The growth rates were determined against a common competitor in isolated cultures. This analysis, in contrast with the nutrient limitation hypothesis, revealed that although haploids grew faster than diploids in rich medium, their fitness was similar in competition. The poor performance of the tetraploids under all conditions suggested that they were of limited use in the investigation of the nutrient limitation hypothesis, also taking into consideration the need for genetic buffering and adaptation to environmental conditions [128]. Another study focusing on an isogenic series was conducted in order to test if cells with higher ploidy levels had higher fitness following mutagenesis with the alkylating agent ethane methyl sulfonate (EMS). Although the fitness reduction in haploids was more apparent than that in diploids, haploids and diploids were shown to recover equally in response to mutagenesis. Tetraploid cells were observed to behave no differently to the diploids. Cells with higher ploidy levels were shown to be more efficient in removing induced mutations, leading to increased growth rates. The presence of EMS was observed to cause both haploids and tetraploids to evolve toward diploidy, which was the ancestral state of *S. cerevisiae.* The reason for this evolution was explained by the fact that diploidy would mask a mutation on a single allele and protect the organism, providing a short-term advantage in terms of fitness. On the other hand, the long-term disadvantage associated with this situation was reported to be that mutations would persist for longer if they were masked from natural selection as they would be in diploids [129].

Gene duplication was previously suggested as an “alternative predictable drive” to increase morphological complexity. The changes in gene content would be via any type of duplication including autotetraploidy, local duplications, and segmental duplications or by any innovation mitigating the dosage effect. Following WGD, tetraploidy was followed by the removal of duplicate genes except for those (such as ribosomal protein genes) for which an increase in gene dosage produced a selective advantage in the sugar-rich environments provided by the emergence of the flowering plants 100 million years ago. Moreover, the balanced gene drive was also proposed as a measure to ensure the enrichment of a genome for regulatory products toward increasing complexity [130]. Polyploidy was previously reported to be very common in nature. Although its incidence would be more useful and better tolerated in lower eukaryotes, unscheduled tetraploidization would be cancer-inducing in more complex organisms and this condition might even result in chromosome instability causing genetic disorders usually followed by premature death. Most cancers were reported to result from a combined association of the presence of tetraploidy and an additional mutation, such as a protein 53 (p53) deficiency or Mad2/Eg5 overexpression. An increase in ploidy was associated with chromosomal rearrangements, translocations, and amplifications and the main reason behind this series of events was associated with an impaired mechanism to deal with DNA damage. This impairment was thought to be associated with the presence of less efficient repair mechanisms in tetraploids, causing the repair process to take longer, or other mechanistic problems associated with repair including the accumulation of an increased amount of spontaneous DNA damage in tetraploids, the accumulation of DNA breaks through mitotic arrest and abnormal mitosis, as well as the high occurrence of DNA breakage caused by aberrant cytokinesis [131].

A genome-wide polyploidy analysis in yeast focused on lethality induced by the deletion of a gene in triploids or tetraploids although the corresponding haploid or diploid deletants were non-lethal. The genome-wide screen identified 39 mutations that were associated with the survival of the tetraploid yeast. Almost all of these genes were shown to affect genomic stability by impairing homologous recombination, sister chromatid cohesion, or mitotic spindle functions. These results led to the suggestion that geometric constraints in tetraploids might have had a role in genome stability and might even lead to disease states including cancer [132]. The quantitative growth assays of 38 stable and fully isogenic aneuploid yeast strains with varying genomic contents between 1N and 3N revealed that gene expression was affected at both the transcriptomic and the proteomic level and would generate significant phenotypic variation to bring about fitness gains under diverse conditions [133]. Another recent study suggested that the cells may use chromosomal duplication as a transient evolutionary solution to stress. Although aneuploidy would often be considered to be a burden on the cell, it was shown to confer a selective advantage under stressful conditions such as a heat shock or a sudden change in pH. The cells used chromosome duplication as a crude solution to these stresses, representing a transient escape route at the individual gene level until more efficient and permanent solutions could be developed. This was suggested as a “quick fix” to maintain survival in response to a strong and abrupt stress. The chromosome duplication was shown to be eliminated even when the stress remained in the environment. Moreover, if the stress was applied gradually, this mechanism is not utilized by the cells at all and alternative solutions were pursued [134].

The effect of gene dosage on several processes in yeast was analyzed through employing tetraploid strains in the studies. Tetraploids were often investigated to see whether an increase in gene dosage would increase flux through a particular pathway and thus aid in understanding and predicting metabolic control. In a very early such study, the effect of gene dosage on galactose utilization in yeast was investigated under both aerobic and anaerobic conditions in an attempt to increase flux through galactose utilization. The effect of gene dosage was investigated comparatively between an isogenic series of tetraploid, diploid, and haploid strains. Galactose oxidation and fermentation were reported to be influenced by gene copy number and the *GAL1* gene was determined to exert a quantitative control over the biosynthesis of galactokinase [135]. Increasing the copy number of genes in a complete pathway was employed as a strategy to increase the in vivo flux through the tryptophan biosynthesis pathway via genetic manipulations. The analysis indicated that increasing the copy number of all the genes in the pathway from chorismate to tryptophan had a more-than-additive effect on the overall flux through the pathway owing to the non-linear response of the enzyme activities. The expression of the genes involved in tryptophan biosynthetic pathways was doubled individually resulting in only very modest improvements in the flux to tryptophan. On the other hand, the doubling in copy number of all five genes in the arm of the pathway from chorismate to tryptophan improved the flux by some 8.8-fold, whereas a 24-fold increase would have been expected according to the coordinate theorem (equivalent to summation theorem), indicating the control of other enzyme steps over the tryptophan biosynthetic flux [136].

Tetraploids were employed in a study by Chi and Liu [137] in order to achieve high alcohol production yields by the hydrolysis of raw ground corn. Higher rates of ethanol production as well as a higher tolerance to ethanol were reported to be achieved using tetraploid strains rather than their haploid parental strains. In a more recent study employing a novel strategy to construct yeasts with improved amylase gene expression, δ-integration and polyploidization were used in conjunction. Following the insertion of coding sequences for an alpha-amylase and a glucoamylase/alpha-agglutinin fusion protein into haploid yeast strains, diploid stains were constructed by mating and later the tetraploid strains were constructed by cell fusion. The ethanol productivity in the fermentation of raw starch was reported to improve with increased ploidy providing a strategy for constructing yeasts that would improve the practical potential of the organism [138]. Gene dosage and varying the copy number of genes was used to establish a logical model of the yeast mitotic cell cycle in a recent study that aimed to identify the genes having the greatest control over cell growth rate; novel tetraploid-specific phenotypes were also identified using this approach (Alcasabas et al., our unpublished data).

Tetraploidy was also employed in a cross-species evolutionary study on postzygotic isolation as a measure in which the degree of sterility in hybrid tetraploids relative to hybrid diploids was used in testing dominant genic incompatibility. Six *Saccharomyces* species were used in the study, in which sterile hybrids of *S. cerevisiae* and five closely related species were made fertile by genome doubling and achieving tetraploidy. This study revealed that hybrid sterility, which was previously thought to be due to deleterious epistatic interactions between genes from different species, had different underlying causes and dominant genic incompatibility either did not make any contribution or could be overcome by tetraploidy in yeast [139].

### 5.4 Genetic interactions, complex genetic interactions, and their effect on growth phenotype

The use of the term epistasis goes back nearly 100 years when Bateson defined it from a biological point of view and Fisher from a statistical point of view. Epistasis was reported as an important component of the genetic architecture of many biological traits for canalization and stabilizing selection. The presence of genetic buffering resulted in the presence of a redundant and robust genetic network architecture [140]. Statistical epistasis allowed researchers to identify genetic interactions quantitatively since it was defined as a deviation from the additivity or multiplicativity in a mathematical model where the relationship between multilocus genotypes and phenotypic variation in a population is not predictable based solely on the actions of the genes considered singly [140]. In the yeast *Saccharomyces cerevisiae*, more than 80% of the genes were reported to be non-essential, indicating the presence of a genomic buffering mechanism against the phenotypic consequences of genetic perturbation. Because of such genetic redundancy, the functions of these genes were reported to remain elusive. The identification of genetic interactions was proposed as a tool to unravel these elusive functions.

#### 5.4.1 Identification of genetic interactions in yeast

Pairs of yeast genes whose double-deletion mutants yielded a synthetic sick or lethal (SSL) phenotype were screened in a high-throughput fashion for the first time using the method of Synthetic Genetic Arrays. The screening of a single query gene, *BMI1* across the whole genome revealed novel relationships between cytoskeletal organization and DNA synthesis and repair. Further analysis of these interacting gene pairs yielded a resulting network of 291 interactions among 204 genes [141]. This methodology was further extended to the analysis of 132 query genes, mainly having a role in actin-based cell polarity, cell wall biosynthesis, microtubule-based chromosome segregation, DNA synthesis and repair, which were screened across approximately 4700 viable deletion mutants and the network constructed by the synthetically sick or lethal pairs comprised nearly 4000 interactions between approximately 1000 genes [142]. This analysis was further extended to attain a complete genetic landscape of the yeast, *Saccharomyces cerevisiae.* A functional map was obtained by evaluating 5.4 million possible gene–gene pairs corresponding to approximately 75% of the yeast genome and genes involved in similar biological processes were observed to cluster in the same subsets. The wiring diagram of pleiotropy in the cell could be observed through the functional cross-connections between bioprocesses [143].

A limited number of studies were also conducted for the identification of genetic interactions in the fission yeast as one of the few non-*cerevisiae* yeast species with which epistasis was studied. The presence of epistatic effects in the control of cell division in fission yeast was investigated as one of the very early efforts in determining genetic interactions in non-*cerevisiae* yeast species [144]. A more recent effort using high-throughput technologies investigated the *Sz. pombe* genetic interaction network and its structure. The epistatic mini-array profile (E-MAP) of the fission yeast was studied and compared with that of *S. cerevisiae* in order to understand how genetic interaction networks had evolved [145].

Identification of genetic interactions in a focused sub-network has been more frequently used by researchers to understand epistasis and genetic variation in yeast as a model organism. Brem and Kruglyak [146] identified QTLs and used this information to predict epistasis and were able to find some evidence for epistatic interactions in 16% of the transcripts that belong to classes of inheritance patterns that underlie genetic variation. Moreover, the investigation of secondary loci interacting with primary QTLs indicated that many QTLs that would be missed by single-locus tests would be detected through two-stage analyses and thus would allow the identification of more interactions [147]. Genetic interactions between TFs were previously reported to cause natural variation in yeast resulting in phenotypic diversity within species. The genetic interactions between genomic regions affecting quantitative traits are of particular interest since TFs were observed to reside in these regions. The analysis of these regions indicated that a small number of nucleotides created complex and quantitative variations in phenotype, highlighting the importance of single-nucleotide polymorphisms (SNPs) in epistasis [148]. A concise review of recent studies on naturally occurring recombinant strains was carried out by Liti and Louis [149], who discussed not only the QTLs themselves but also the interaction between different QTLs, and between QTLs and the environment.

Synthetic genetic interactions were used to construct an essential synthetic genetic network using temperature-sensitive conditional alleles. The genetic interaction network of the 286 essential genes was determined to have five times the interaction density of that of the non-essential synthetic genetic network with a total of 386 interactions and the network was also determined to be biased toward interactions between genes that shared common Gene Ontology Process annotations [150]. Another study focusing on the interactions between deleterious mutations, by randomly crossing mutants with growth defects and assaying the growth of the progeny, indicated the presence of positive epistatic effects. The study suggested that epistasis diminished the negative effects of the individual mutations since the ability to grow at high rates contributed significantly to fitness [151].

The impact of epistasis in understanding the genetic basis of natural variation was discussed previously in detail. There is limited evidence that epistatic variance has an important role in most populations although, at the cellular level, epistasis at multiple loci was reported to make more significant contributions to additive variance [152]. The possibility of epistasis between the mitochondrial and nuclear genomes of yeast was investigated in two yeast genotypes one descendant that evolved asexually and independently from a known ancestor and its ancestor and another analysis was carried out using virulent and non-virulent *S. cerevisiae* strains. The study revealed the contribution of mitochondrial genome to progeny fitness as well as the presence of significant epistasis between the mitochondrial and nuclear genomes [153]. The adaptation of organisms to divergent environments was studied by testing key predictions of speciation theory using yeast populations. The evolution of reproductive isolation was shown to proceed through a gain of fitness by antagonistic epistasis during adaptation [154]. Musso et al. [155] investigated condition-dependent epistasis among whole-genome duplicates in a study where the key focus was on the epistasis among 399 paralogous pairs of metabolic enzymes. The high functional overlap among the whole-genome duplicates in yeast indicated that, although evolutionarily unfavorable, functional redundancy was not lost. More than a third of the pairs were determined to be epistatic under standard laboratory conditions whereas this ratio was shown to increase when stress conditions such as ionic, osmotic, or high-pH stresses were induced.

#### 5.4.2 Genetic interactions as a tool for revealing functional relationships in the cell

The identification of genetic interactions was used as a tool to reveal the functional relationships between and within protein complexes and a case study was conducted on yeast chromosome biology using E-MAPs involving 743 genes. The identification of epistatic interactions was used to identify and dissect functional multi-protein complexes and to organize them into distinct pathways [156]. In another study, the genes having a role in chromosome stability were identified using synthetic lethal (SL) and synthetic dosage-lethal screens of kinetochore mutants and a total of 211 non-essential genes were identified that were unable to tolerate defects in kinetochore function [157].

In order to achieve a deeper systems-level understanding of the regulation of gene expression, the general transcription factors (GTFs), and site-specific DNA-binding transcription factors (STFs) were investigated by quantitative genetic profiling using E-MAP. The global epistatic patterns indicated that the preference for positive genetic interactions among GTFs and negative genetic interactions among STFs, which are most frequently observed among non-essential genes having roles in redundant pathways. The study indicated that the parallel and compensating relationships between regulators, rather than linear pathways, were observed to characterize transcriptional circuits [158]. The genetic interactions between 38 query genes that are members of the histone acetylation/deacetylation complex were investigated using diploid-based synthetic lethality analysis on microarrays. Systematic analysis of the genetic interactions revealed new characterizations of the histone deacetylation complex and the essential nucleosome acetyltransferase of histone 4 (H4) complexes thus illuminating mechanisms of intricate cellular processes [159]. Vizeacoumar et al. [160] described a novel screening approach combining synthetic genetic arrays and high-content screening in order to explore yeast spindle morphogenesis and reported this novel approach to be a powerful and practicable tool for identifying cellular functions in any pathway.

#### 5.4.3 In silico prediction of epistasis

The laborious nature of the experimental identification of genetic interactions led researchers toward the prediction of epistasis from information available in different layers of biological network structures. One of the earlier efforts focused on the prediction of synthetic sick/lethal pairs in *S. cerevisiae* using probabilistic decision trees on an integrated network of multiple types of data including localization, mRNA expression, physical interactions, protein function, and characteristics of network topology [161]. Another study suggested graph theory as a powerful tool to study the network basis of pleiotropy and epistasis, two exceptions to the Mendelian one-gene-one phenotype paradigm [162]. Epistatic interactions among the genes constituting a metabolic network were studied using flux balance analysis to identify unexpected changes in fitness in double knockouts of 890 metabolic genes in *S. cerevisiae.* The buffering, aggravating, and non-interacting gene pairs were identified and the interaction network could be organized into monochromatic, function-enriched modules, thus extending the concept of epistasis from gene pairs to functional units [163]. He et al. [164] focused on in silico mapping of the positive and negative epistasis in the *S. cerevisiae* metabolic network using flux balance analysis. They concluded that negative epistasis was more prevalent within the genes whose gene products catalyze non-essential biochemical reactions with overlapping functions, whereas positive epistasis was more frequent between genes involved in essential reactions but that did not have overlapping functions. Sanjuán and Elena [165] discussed the concept that simpler genomes had been shown to display antagonistic epistasis whereas more complex genomes evolved toward displaying synergistic epistasis. The proposed reason for such an effect was that antagonistic epistasis would dominate compact genomes with few non-pleiotropic biological functions whereas synergism would have been a result of the mutational robustness of the complex genomes [165]. In a recent study by Park and Lehner [166], an SSL interaction network was integrated with gene expression profiles to identify the relationship between the connectivity of the genes in the epistatic network and the variation in their expression levels. The results of this analysis indicated that highly connected hubs had more stable expression profiles across a range of environmental conditions and this had been a constraint on evolution.

The effect of plasticity, and hence the condition-dependence, of genetic interactions was investigated through a systems-level in silico flux balance analysis in yeast and the findings were verified through in vivo gene-deletion studies. The results of the study indicated that redundant genes were determined to have significant fitness contributions under specific environmental conditions and that they were required for environmental adaptation. Nearly 50% of the SL interactions identified in the metabolic network were restricted to only one or two of the environmental conditions that were under investigation whereas only 14% were detected under all conditions [73]. The effects of environmental stress and genetic alterations on the selection of new mutations were discussed by Agrawal and Whitlock [167] from an evolutionary perspective, putting a strong emphasis on fitness. Different types of stress were considered to affect selection differently and it was also noted that, if a population was displaced away from the optimum because of environmental or genetic stress, there would be an increased opportunity for beneficial mutations.

The collection of information on genetic interactions has allowed researchers to interpret the data for gene function prediction but, at the same time, has revealed the need to develop more systematic methodologies to quantify and interpret large-scale genetic interaction data. Ye et al. [168] predicted functions for several genes in yeast using the information provided by the SL gene pairs. An in silico approach used SL gene pairs to bridge pathways and to develop a “parallel pathway model” in which they could successfully predict inferences regarding the membership of protein complexes [168]. Several studies focused on methodologies for accurate identification and quantification of the extent of epistatic interactions. An early attempt to account for the continuum of genetic interaction strengths specifically addressed data from synthetic genetic arrays and E-MAPs. A methodology was developed in order to account for the intrinsic measurement errors, the determination of the confidence with which the genetic interaction could be assigned, and the functional relationships between pairs of genes [169]. Another study focused on an elaborate methodology to correctly quantitate fitness. This approach attempted to account for several problems that were encountered when colony size on solid medium was used as a proxy for the measurement of growth and thus fitness. The experimental sources of variation were identified and normalization strategies developed for achieving accurate measurements of fitness in order to be able to obtain high reproducible results [170].

Completion of the genetic landscape of a eukaryote for the first time facilitated research toward understanding the genetic architecture of growth traits and the implications of pleiotropy through the reconstruction of a global map of epistasis. For this purpose, epistatic interaction information that was collected from 354 different conditions were compiled together to obtain the global map of epistasis. Increased epistasis between genes underlying the same growth trait in yeast was determined not to be as important as expected. In contrast, the epistatic network structure revealed that the hubs tended to epistatically interact with each other more frequently than expected. The network hubs were determined to have higher effect on phenotype, and hence on pleiotropy, which might imply that pleiotropic genes would have higher chances of developing functional overlaps among themselves in the fixed functional space of a cellular system [171]. Another study focused on the genetic interactions among the metabolic gene pairs. The distributions of positive and negative interactions predicted from the yeast metabolic network and empirically confirmed, were investigated using functional modularity and flux coupling analyses. A mechanistic explanation providing a link between the degree of genetic interactions, pleiotropy, and gene dispensability was provided by this study [172].

Efforts to study epistatic effects were not only limited to the investigation of haploid double-deletion mutants to unravel unexpected outcomes in fitness, and hence reveal the presence of an epistatic effect. Pairing heterozygous mutant loci at random and testing for epistasis was conducted on yeast strains, in which rare and random mutations were induced by ethyl methanesulfonate (EMS) in a study conducted by Szafraniec et al. [173]. Although the decrease in the fitness of homozygotes carrying a single mutation was approximately 2%, the harmful effects of these mutations were reduced in the heterozygotes by more than fivefold. The fitness of the mutants bearing double heterozygous mutations was reported to decrease by less than half a per cent on average, indicating a very mild and weak form of epistasis in the heterozygous loci regardless of how harmful the mutations were in haploids or homozygous diploids [173].

The only comprehensive study on complex (i.e. doubly heterozygous) genetic interactions in *S. cerevisiae* was conducted using the yeast gene specifying actin, *ACT1*, as the query gene. The complex hemizygous strains that were constructed were screened for complex haploinsufficient interactions with actin and 208 such pairs were identified. The analysis of the data suggested that the loss of binding capability of actin-binding proteins was the cause of the complex haploinsufficiency that was observed [174]. The first large-scale screen on *Candida albicans* was conducted to identify complex haploinsufficiency in the genetic network in order to understand the morphogenic transition between yeast and the filamentous growth in this human fungal pathogen. The relationship between morphogenesis and pathogenesis was investigated using a heterozygous mutant *CBK1*/*cbk1*, a gene encoding a key regulation of Ace2p activity and cellular morphogenesis (RAM) pathway protein kinase, and screening it against the remaining heterozygous mutants. Decreased filamentation was used as a measure of insufficiency in the doubly heterozygous mutants. The results indicated the necessity for the presence of a balance between the activities of the protein kinase A (PKA) and RAM pathways for maintaining normal morphogenesis [175].

## 6 Concluding remarks

Our understanding of the control of yeast growth may soon enable us to improve the organism’s production performance by increasing its maximum rate of growth or uncoupling the generation of a commercially valuable product from that of biomass. In all, our increasing ability to predict complex genotype–phenotype interactions should enable us to more effectively engineer yeast as a cell factory and to use it as a living model of human or pathogen cells in intelligent screens for new drugs.

## Abbreviations

DNA: deoxyribonucleic acid
E-MAP: epistatic mini-array profile
EMS: ethane methyl sulfonate
GO: Gene Ontology
MCA: metabolic control analysis
mRNA: messenger RNA
PET: nuclear petite
PKA: protein kinase A
QTL: quantitative trait locus
RNA: ribonucleic acid
SL: synthetic lethal
TF: transcription factor
WGD: whole-genome duplication
YSBN: Yeast Systems Biology Network
